# Tensile Response and Energy Absorption of Galvanized Steel Mesh-Reinforced Cement Mortar with Alkali-Resistant Glass Fibers

**DOI:** 10.3390/ma19163491

**Published:** 2026-08-18

**Authors:** Leonardo Rodríguez, Rodrigo Valle, César Garrido, Marian Valenzuela, Víctor Tuninetti, Felipe Núñez

**Affiliations:** 1 Construction Multidisciplinary Research Group, Facultad de Arquitectura, Construcción y Medio Ambiente, Universidad Autónoma de Chile, Talca 3460000, Chile; leonardo.rodriguez1@cloud.uautonoma.cl (L.R.); rodrigo.valle@uautonoma.cl (R.V.); 2 Department of Mechanical Engineering, Universidad del Bío-Bío, Concepción 4081112, Chile; cgarrido@ubiobio.cl; 3 Doctoral Program in Sciences of Natural Resources, Universidad de La Frontera, Casilla 54-D, Temuco 4780000, Chile; m.valenzuela16@ufromail.cl; 4 Sustainable Building Design Laboratory, Department UEE, Faculty of Applied Sciences, University of Liège, 4000 Liege, Belgium; 5 Department of Mechanical Engineering, Universidad de La Frontera, Temuco 4811230, Chile

**Keywords:** fiber-reinforced mortar, hybrid reinforcement, direct tensile testing, energy absorption capacity, thin cementitious composites, post-cracking behavior

## Abstract

**Highlights:**

- Alkali-resistant glass fibers substantially enhance mortar tensile performance.
- Crosshead-derived apparent tensile stiffness increased 10.43-fold.
- The 0.2% offset stress and ultimate tensile strength improve by 154% and 74%, respectively.
- Apparent post-offset energy absorption improves, boosting energy absorption by 68%.

**Abstract:**

This study investigates the direct tensile mechanical behavior of cement mortar plates reinforced with a galvanized steel mesh and randomly incorporated alkali-resistant glass fibers. An experimental program was executed using direct tensile tests on thin mortar specimens containing fiber volumetric fractions of 0%, 4%, 6%, 8%, and 10% relative to the cement volume. To rigorously characterize the mechanical response, the study quantified the apparent initial stiffness, 0.2% offset stress, ultimate tensile strength, and post-offset energy absorption capacity. Results indicate that increasing alkali-resistant glass-fiber content systematically modified the global tensile response of the composite system. At 10% glass-fiber content, the mean crosshead-derived apparent initial tensile stiffness was 10.43 times that of the reference group without glass fibers. The characteristic stress determined using the adopted 0.2% offset criterion and the ultimate tensile strength increased by 154.5% and 74.1%, respectively, while the apparent post-offset energy absorption increased by 68.4%. Because strain was derived from crosshead displacement, the apparent stiffness and energy-absorption parameters represent the global specimen–grip–machine response rather than intrinsic material properties. The experimental results exhibited acceptable repeatability, although the apparent tensile stiffness showed greater variability than the strength-related parameters. These findings support the continued development of the investigated composite configuration for thin cementitious elements requiring improved tensile response and damage tolerance.

## 1. Introduction

Cement-based materials are widely used in construction due to their versatility, ease of application and compatibility with masonry and concrete substrates [1,2]. However, their low tensile strength and brittle behavior make them particularly vulnerable to cracking, compromising their durability, serviceability, and long-term performance [3,4]. These limitations are especially critical in thin cementitious elements, plaster layers, repair mortars and precast systems, where tensile stresses determine crack initiation and propagation [5,6]. Therefore, improving the tensile response and post-cracking behavior of cement mortar remains a key challenge in the development of more reliable and durable cement-based materials [7,8].

Cement mortar is inherently a brittle material with very limited tensile strength and deformation capacity [9]. Due to the absence of coarse aggregates, tensile stresses are mainly resisted by the cementitious matrix, leading to early microcrack formation and sudden fracture once cracking has started [10]. While the presence of fine aggregates improves workability and surface finish, it does not significantly improve tensile strength or post-cracking behavior [11]. As a result, cement mortar exhibits poor crack control and limited energy dissipation under tensile loading, prompting the development of reinforcement strategies aimed at improving its tensile performance and structural reliability [12]. One of the most widely used approaches to overcoming the tensile weakness of cement mortar is the incorporation of discrete fibers into the cementitious matrix [13,14,15]. Fiber reinforcement improves tensile performance primarily through crack bridging mechanisms, which delay crack initiation, restrict crack opening, and promote progressive damage rather than sudden failure [16,17]. Among the different types of fibers investigated—including polymer, basalt, carbon, and glass fibers—alkali-resistant glass fibers have become a particularly attractive solution for mortar applications [18,19]. Their high tensile strength, low density, corrosion resistance, and excellent cost–performance ratio make them well suited for thin cementitious elements and prefabricated systems [20,21]. However, conventional glass fibers are susceptible to chemical degradation in the highly alkaline environment of cementitious matrices (pH 12–13), where silica reacts with alkalis and progressively loses its mechanical integrity [22]. The durability of glass-based reinforcement can be adversely affected by the highly alkaline environment of cementitious matrices, motivating the use of alkali-resistant glass-fiber formulations [23].

Most studies on fiber-reinforced cementitious materials have focused primarily on concrete [24,25,26], rather than mortar [27,28,29], with an emphasis on compressive strength, flexural behavior, durability and shrinkage control. These studies consistently demonstrate that fiber incorporation can improve crack control and mechanical performance [30,31]. However, tensile behavior, particularly under direct tension, has received limited attention, especially in alkali-resistant glass fiber reinforced cement mortars. Research has shown that steel and carbon fibers can improve selected mechanical properties of concrete [32,33], while noncorrosive carbon-fiber textile reinforcement can enable thinner structural elements and reduced concrete cover [34]. Other fiber types, such as cotton straw and expanded polystyrene, have been shown to reduce compressive strength while improving flexural performance, highlighting the relationship between stiffness and ductility as a function of fiber type and dosage [35]. Hybrid fiber systems combining polyvinyl alcohol (PVA), steel or basalt fibers have also been explored, showing improved tensile and compressive performance after thermal exposure. PVA–steel combinations offer the most favorable results [36]. While these studies provide valuable insights, they focus primarily on concrete and indirect tensile loading conditions. Additional studies further confirm these trends. For example, Guo et al. [37] demonstrated that increasing the glass fiber content improves flexural toughness and crack resistance in cementitious composites, although tensile behavior under direct loading was not addressed. Gao et al. [38] highlighted that fiber-matrix interfacial bonding critically influences crack bridging efficiency and energy dissipation, particularly in fine-grained cementitious matrices. Similarly, Zhou et al. [39] reported that glass fiber reinforcement significantly increases toughness and post-cracking deformation capacity, however, direct tensile tests were not systematically performed.

Several studies on glass fiber-reinforced cement mortars have reported promising improvements in both mechanical performance and durability. Chen et al. [40] reported that alkali-resistant glass fibers increased the flexural strength of cement-based mortar by up to 57% under the investigated conditions, while glass-fiber powder also reduced autogenous and drying shrinkage. Similarly, Xin et al. [41] demonstrated that recycled glass fiber-reinforced plastic powders, particularly when combined with silica fume, improve matrix densification and fiber-matrix interaction, resulting in strength increases of approximately 40%. In parallel, Xiaochun et al. [23] demonstrated that alkali-resistant glass fibers can be successfully used in demanding applications such as road pavements, provided that durability issues related to alkali corrosion are adequately controlled by complementary cementitious materials. The tensile response of fiber-reinforced mortars has been explored less thoroughly. Fenu et al. [42] reported that glass fibers significantly improve energy absorption capacity under high strain rate loads. These findings highlight the potential of glass fibers to improve post-cracking behavior and toughness; however, systematic studies under quasi-static direct tensile loads remain scarce. Natural fibers and hybrid reinforcement strategies have also been investigated as sustainable alternatives. While natural fibers can improve tensile strength and toughness through crack bridging mechanisms, their long-term durability in alkaline environments remains a significant limitation [43]. Hybrid systems combining natural and glass fibers or recycled rubber particles show that mechanical performance is highly dependent on fiber type, interfacial bonding, and dosage, and that synergistic effects are only observed under carefully optimized conditions [44,45]. These studies further highlight the importance of fiber selection and dosage optimization in cement-based composites.

In addition to conventional fiber-reinforced mortars, several cementitious composite systems have been developed to enhance tensile performance through different reinforcement strategies. Glass fiber-reinforced cement (GRC) and glass fiber-reinforced concrete (GFRC) have been extensively employed in thin prefabricated elements, where randomly dispersed alkali-resistant glass fibers contribute to crack control, toughness, and post-cracking behavior [46]. Alternatively, ferrocement systems consist of one or more layers of continuous steel wire mesh embedded within a cement mortar matrix, in which the mesh provides the primary tensile reinforcement and the mortar acts as the binding medium. This reinforcement strategy promotes the formation of multiple fine cracks, enhances flexural performance and ductility, and increases the load-carrying capacity of thin cementitious elements [47]. More recently, textile- and fabric-reinforced cementitious matrix (TRM/FRCM) composites have attracted considerable attention for structural strengthening applications using continuous glass, carbon, basalt, or polyparaphenylene–benzobisoxazole (PBO) textiles embedded in inorganic mortars [48]. Studies combining continuous reinforcement with discrete short fibers provide the closest conceptual comparison with the present configuration. Zhou et al. [49] investigated carbon textile-reinforced mortar containing dispersed short steel fibers under uniaxial tension and reported that the short fibers improved the tensile response and utilization of the continuous textile reinforcement. Deng et al. [50] similarly showed that incorporating short polyvinyl alcohol fibers into carbon textile-reinforced mortar modified the cracking and failure response and increased tensile strength and strain. More recently, Wang et al. [51] evaluated thin sprayed mortar plates reinforced with directional woven grids, non-directional short polyvinyl alcohol fibers, or their combination under direct tensile and flexural loading. These studies show that continuous and discrete reinforcement systems can be combined at different length scales.

Beyond cementitious systems, recent research on hybrid materials has shown that combining different reinforcing phases can be an effective strategy for adjusting stiffness, deformation, damage tolerance, and energy dissipation. In particular, studies incorporating lightweight cellular polymer phases within reinforcing matrices have shown that the interaction between the structural phase and the surrounding matrix can significantly influence the overall mechanical response. Garrido et al. [52] demonstrated that incorporating a cellular polymer phase into a polyester matrix can improve structural stiffness while maintaining the energy absorption capacity of the underlying structure. This concept was subsequently extended through the evaluation of different reinforcing matrices, showing that the mechanical response of the resulting composite depends largely on the interaction between the load-bearing phase and the surrounding material [53]. More recently, it has been shown that the incorporation of flexible polyurea phases into fiber-reinforced polymer systems modifies failure mechanisms, reduces brittle fracture, and improves damage tolerance under mechanical and impact loads [54,55]. These studies provide extensive evidence that combining reinforcing phases with distinct mechanical functions can promote complementary load transfer and modify damage evolution in heterogeneous materials. This concept is particularly relevant for cement mortar systems that incorporate continuous and dispersed reinforcement, where steel mesh and randomly distributed glass fibers can play complementary roles in load transfer and post-cracking deformation.

To address this fundamental gap, this study executes a systematic macro-mechanical investigation into the direct tensile response of galvanized steel mesh-reinforced cement mortar plates modified with varying contents of alkali-resistant glass fibers. The central hypothesis posits that incorporating high-strength glass fibers into the cementitious matrix, without intentionally controlling their orientation, may modify the load-transfer mechanisms within the steel mesh-reinforced composite, potentially promoting a more uniform stress distribution and improving the overall damage tolerance of the material. To test this premise, the effects of fiber content—ranging from 0% to 10% by cement volume—on the apparent initial stiffness, 0.2% offset stress, ultimate tensile strength, and apparent post-offset energy absorption are quantitatively evaluated under direct tension.

By evaluating these macro-mechanical response parameters, this research provides experimental evidence of the association between alkali-resistant glass-fiber content and the damage tolerance and energy dissipation capacity of cement mortars, supporting the optimization of thin cementitious structural elements. Subsequent sections detail the displacement-controlled direct tensile testing methodology (Section 2), provide a comprehensive physical and statistical analysis of the stress–strain behavior and energy absorption metrics (Section 3), and summarize the structural implications alongside directions for future research (Section 4).

## 2. Materials and Methods

This section details the constituent materials, specimen fabrication protocols, and experimental setups used to evaluate the tensile behavior of cement mortar plates reinforced with galvanized steel mesh and alkali-resistant (AR) glass fibers. The experimental matrix systematically assesses the influence of fiber dosage on apparent initial stiffness, 0.2% offset stress, ultimate tensile strength, and energy absorption capacity.

### 2.1. Fabrication and Configuration of Composite Mortar Specimens

**Figure 1:**
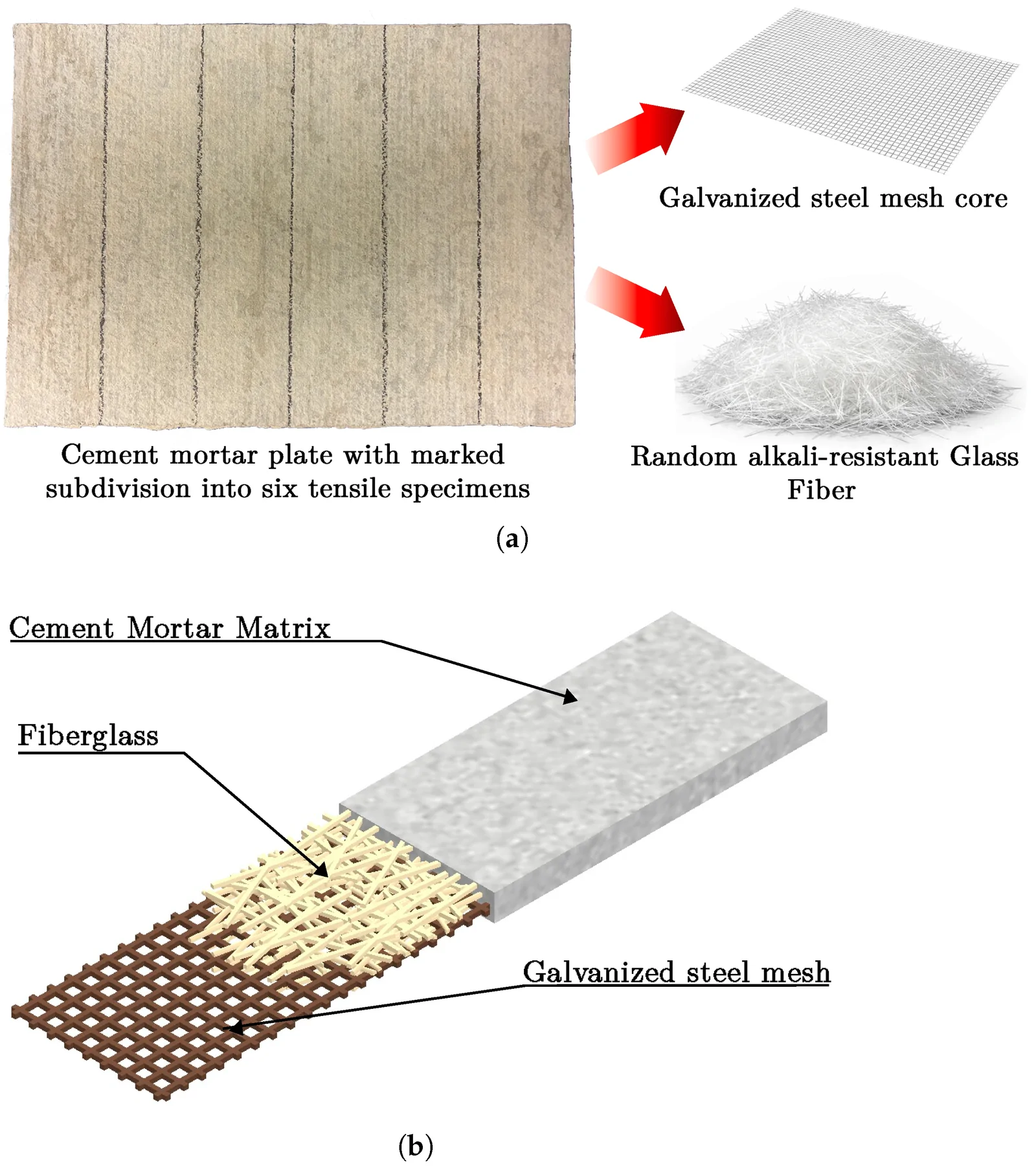
Composite layout and specimen configuration for the tensile tests. Subfigures (**a**) illustrate the plate geometry, specimen subdivision, and reinforcement distribution, while (**b**) shows a 3D layered schematic of the cement matrix, glass fibers, and steel mesh.

Thin cement mortar plates were fabricated with nominal dimensions of 150×225×6 mm (Figure 1a), a geometry selected to represent thin cementitious panels and to enable the evaluation of their mechanical properties through direct tensile tests. The cementitious matrix was prepared using pozzolanic cement, obtained by the co-grinding of clinker, pozzolan, and gypsum. The cement is classified as a standard-grade pozzolanic cement according to NCh 148 (Chilean Standard) which incorporates requirements consistent with ASTM C150 [56]. The mortar contained 500 g of cement, 500 g of each sand fraction, and 250 g of potable water, corresponding to a water-to-cement mass ratio of 0.50.

The fine aggregate was classified by sieve analysis [57] into three particle-size fractions: coarse, medium, and fine. The coarse fraction corresponded to the material passing through the ASTM No. 4 sieve (4.75 mm) and retained on the ASTM No. 8 sieve (2.36 mm); the medium fraction consisted of particles passing through the ASTM No. 8 sieve and retained on the ASTM No. 16 sieve (1.18 mm); and the fine fraction corresponded to the material passing through the ASTM No. 30 sieve (0.60 mm). Subsequently, the aggregate was cleaned by sieving through the ASTM No. 200 sieve (0.075 mm) to remove detrimental fine particles, mainly silt and clay.

A galvanized welded steel mesh with a wire diameter of 1.5 mm and a square opening of 12.7×12.7 mm (Figure 1b) was centrally embedded in each plate to provide reinforcement and ensure structural stability during handling and testing. Representative mechanical properties reported in the literature for galvanized welded steel meshes include a Young’s modulus of 200 GPa, an offset stress of 550 MPa, and an ultimate tensile strength of 600 MPa [58].

Commercially available alkali-resistant (AR) glass fibers were randomly incorporated into the cementitious matrix at volumetric fractions of 0%, 4%, 6%, 8%, and 10% relative to the cement volume (Figure 1b). The fibers were supplied as discrete filaments with a nominal length of 36 mm and a filament diameter of 19 μm, resulting in an aspect ratio of approximately 1895. Typical mechanical properties reported in the literature include a Young’s modulus of 75 GPa, a tensile strength of 1700 MPa, and a density of 2.70 g/cm^3^ [59]. The fibers were manually incorporated into the fresh mortar during mixing, without any intentional alignment or orientation. Consequently, the manufacturing procedure resulted in random incorporation of the fibers, although their actual orientation within the specimens was not quantitatively characterized. The steel mesh was positioned at mid-thickness, bounded by symmetric layers of fiber-reinforced mortar on both faces.

After fabrication (Figure 1a), the plates were kept inside the molds for the first 24 h before demolding. Subsequently, the specimens were cured for 28 days under laboratory environmental conditions prior to direct tensile testing. The temperature and relative humidity during curing were not systematically recorded.

Table 1 summarizes the experimental groups and their corresponding glass fiber contents. Each group was defined according to the volumetric percentage of alkali-resistant glass fibers incorporated into the cement mortar matrix, namely 0%, 4%, 6%, 8%, and 10%. For each fiber content, six tensile specimens were tested, providing a consistent number of specimens for within-plate comparative analysis. This experimental design enabled the mechanical response of each group to be evaluated based on representative average values while accounting for the inherent variability associated with the random incorporation of glass fibers within the cementitious matrix. The required fiber mass was calculated from the specified volumetric content relative to the cement volume, considering cement and AR-glass fiber densities of 3.15 g/cm^3^ and 2.70 g/cm^3^, respectively.

**Figure 2:**
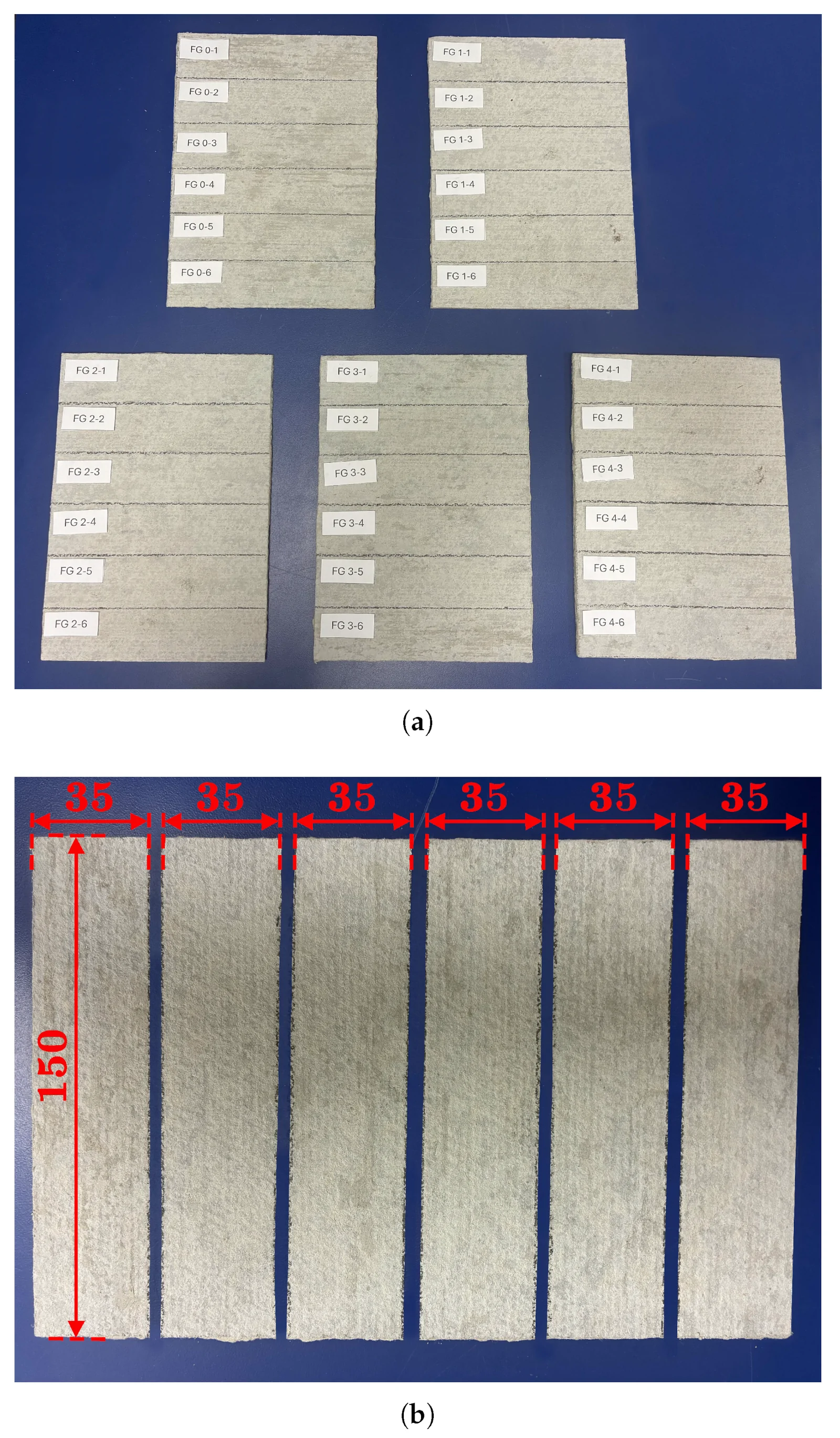
(**a**) Identification of the specimens cut from each parent mortar plate and (**b**) nominal specimen geometry and dimensions. All dimensions are in millimeters.

To guarantee identical production and curing history within each experimental group, one monolithic plate was cast for each of the five fiber fractions (0%, 4%, 6%, 8%, and 10%) (Figure 2a). Each cured plate was subsequently partitioned into six prismatic tensile specimens with nominal dimensions of 150×35×6 mm (Figure 2b). A kerf material loss of approximately 3 mm per cut was factored into the initial plate design to ensure uniform post-subdivision specimen geometries.

This extraction protocol provided six tensile specimens per fiber-content level, as summarized in Table 1. Because all six specimens at a given fiber content were cut from the same parent plate, they characterize specimen-to-specimen variability within a single manufacturing batch rather than variability between independently manufactured batches. The tensile results reported for each experimental group therefore represent the average monotonic response of these six within-plate specimens, while differences among fiber-content groups are interpreted as comparative trends within the present experimental program.

### 2.2. Direct Tensile Testing Procedure

**Figure 3:**
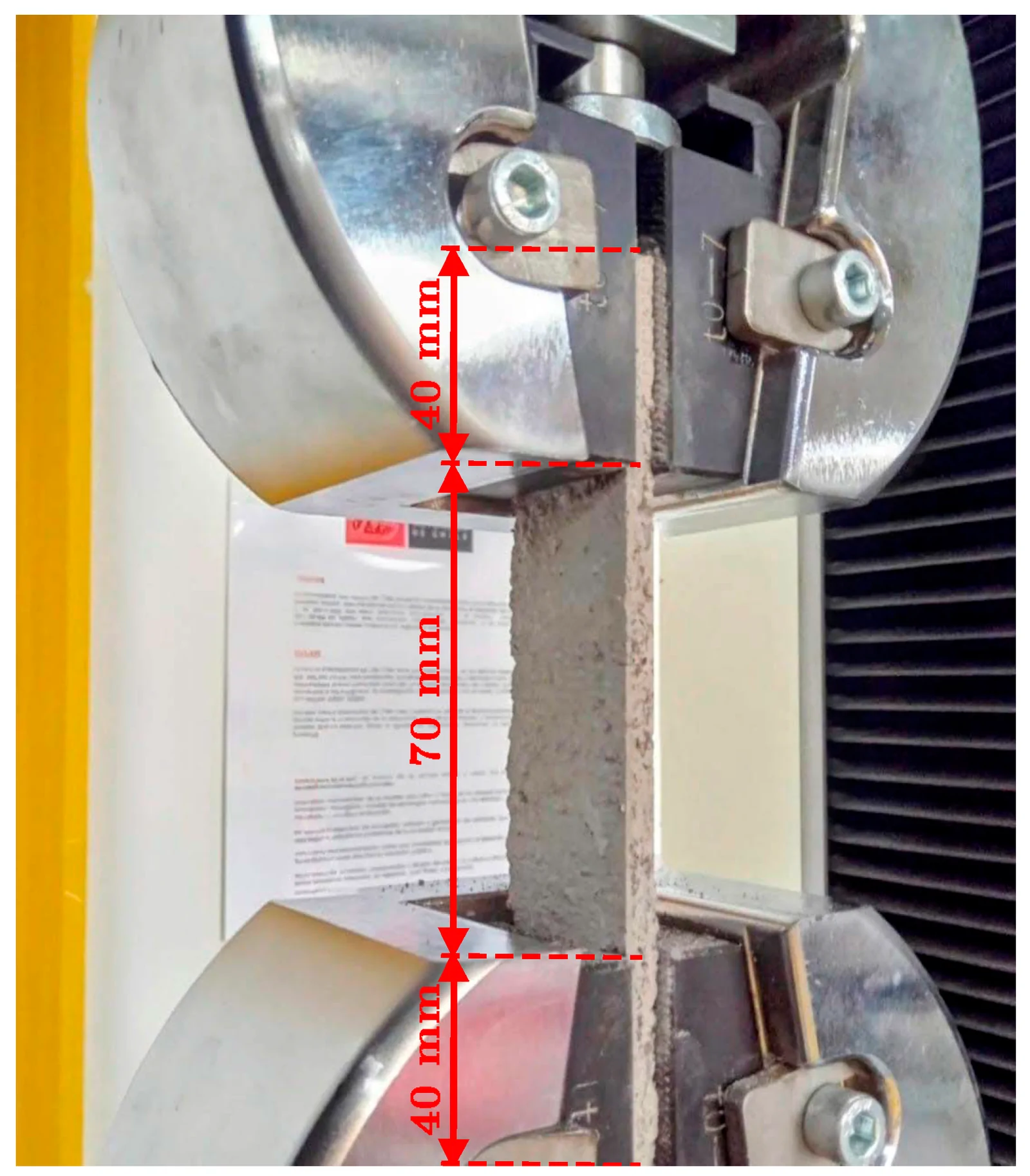
Direct tensile test arrangement showing the universal testing machine, serrated mechanical grips, gripped regions, 70 mm nominal gauge length, and position of the embedded galvanized steel mesh.

Direct tensile testing was conducted to characterize the uniaxial tension response and macro-mechanical behavior of the composite mortar specimens. Monotonic direct-tensile tests were performed using a Laryee NE-34100 universal testing machine equipped with a 100 kN load cell (Laryee Instruments, Xicheng District, Beijing, China). Prismatic specimens with nominal dimensions of 150×35×6 mm were mounted securely within the machine gripping jaws. Testing was performed under displacement control at a constant crosshead speed of 2.0 mm/min until ultimate material failure. The setup of the tensile test is shown in Figure 3. The specimens were secured using mechanical grips equipped with serrated surfaces to increase friction at the contact interface and prevent slippage during load application. The same clamping procedure was applied consistently to all specimens. The distance between the grips for all specimens, defined as the gauge length ($L_{0}$), was 70 mm. During testing, the specimen–grip interfaces and the gripped regions were visually monitored for gross relative displacement, localized mortar crushing, and premature fracture. The visual observations did not constitute a systematic crack-mapping or failure-mode characterization procedure; consequently, crack initiation locations, crack trajectories, and final failure zones were not consistently documented for all specimens. No independent displacement measurement was installed at the specimen–grip interfaces; consequently, minor seating or slip effects could not be separated from the crosshead displacement and are included in the reported global strain-based response parameters.

Given the absence of specialized standardized testing methods for thin cementitious panels reinforced with a hybrid steel mesh and randomly incorporated fibers, ASTM D3039/D3039M [60] was adopted as an engineering reference. This protocol strictly governed the specimen gripping mechanism, spatial loading configuration, and displacement-rate control. Throughout each test, the integrated data acquisition system automatically logged the continuous history of the applied force (*P*) and crosshead displacement (δ) to construct characteristic engineering profiles for each replicate.

Mechanical parameters evaluated to quantify the composite performance included apparent initial stiffness, 0.2% offset stress, and ultimate tensile strength. Engineering stress (σ) was calculated using Equation (1):

(1) $$\sigma =\frac{P}{A_{0}}$$

where $A_{0}$ represents the initial nominal cross-sectional area derived from the measured specimen width and thickness. Engineering strain (ε) was computed from the logged crosshead displacement relative to the initial gauge distance between the gripping boundaries:

(2) $$\varepsilon =\frac{\delta }{L_{0}}$$

Because the deformation data stem directly from crosshead stroke records rather than an attached extensometer, the reported values represent global engineering strains associated with the coupled specimen–machine framework. Consequently, the elastic metrics reported herein are defined as apparent tensile stiffness values to differentiate them from the true intrinsic Young’s modulus of the cementitious matrix. Apparent initial stiffness was mathematically isolated from the highly linear early-stage elastic regime of each calculated stress–strain (σ−ε) curve.

To ensure a reliable determination of the apparent initial stiffness, the calculation was limited to the initial linear portion of the stress–strain response prior to the onset of nonlinear behavior. The slope of the (σ−ε) curve within this initial linear region was calculated using a linear regression method, minimizing the influence of local material heterogeneities.

A characteristic stress was also determined for each specimen using the conventional 0.2% strain criterion. Since cementitious materials exhibit a predominantly quasi-brittle response and do not have a well-defined yield point, this criterion was adopted solely as a reproducible engineering procedure to objectively identify a characteristic point on the stress–strain curve. Specifically, a line parallel to the initial linear portion of the curve (σ−ε) was shifted horizontally by 0.2% strain, and its intersection with the experimental stress–strain curve was defined as the 0.2% strain stress. This procedure was used solely to ensure that the same objective criterion was consistently applied to all specimens, thereby allowing a direct comparison between the different glass fiber contents. The selected point should not be interpreted as evidence of a true material yield point or as an intrinsic property of the cementitious composite material.

Finally, the maximum tensile strength was defined as the maximum stress reached during the tensile test before the specimen broke. This parameter reflects the combined contribution of the cement matrix, the glass fiber reinforcement, and the steel mesh to the load-bearing capacity of the composite system. All mechanical properties of each specimen were evaluated and subsequently analyzed as a function of glass fiber content. This comprehensive evaluation allowed for a systematic investigation of the influence of alkali-resistant glass fiber incorporation on the apparent initial stiffness, 0.2% offset stress, and ultimate tensile strength.

### 2.3. Apparent Post-Offset Energy Absorption

In addition to the stiffness- and strength-related properties described in the previous section, the apparent post-offset energy absorption of the cement mortar composites was evaluated. This parameter is relevant for the comparative assessment of fiber-reinforced cementitious materials, because the incorporation of discrete fibers may modify their tensile response through mechanisms consistent with crack bridging, stress redistribution, and progressive damage evolution. At the macro-scale, enhanced energy-absorption responses have also been investigated through alternative inclusions, such as 3D-printed polymeric auxetic lattices embedded within metamortars [61]. Furthermore, the incorporation of sustainable industrial by-product fibers has been reported to improve tensile and crack resistance in compressed cementitious and earth matrices [62]. Accordingly, the area under the global stress–strain response provides a comparative measure of the post-offset tensile behavior of the investigated composite configurations.

The apparent post-offset energy absorption was calculated from the σ–ε response obtained during the direct tensile tests. Specifically, it was defined as the area under the apparent stress–strain curve from the point determined using the adopted 0.2% offset criterion to the end of the recorded response. This approach enabled a systematic comparison of the influence of glass-fiber content on the global post-offset tensile response beyond conventional strength-based parameters. Thus, the apparent post-offset energy absorption was calculated using Equation (3):

(3) $$E_{a}=\int_{\varepsilon_{0.2}}^{\varepsilon_{\mathrm{end}}}\sigma \left(\varepsilon \right)\;d\varepsilon$$

where $E_{a}$ denotes the apparent post-offset energy absorption, σ(ε) represents the engineering stress as a function of the apparent global axial strain, $\varepsilon_{0.2}$ corresponds to the strain at the intersection with the adopted 0.2% offset line, and $\varepsilon_{\mathrm{end}}$ is the strain at the end of the recorded tensile response. Because strain was determined from crosshead displacement, the resulting stress–strain response includes not only specimen deformation but also the compliance of the testing machine and gripping system, together with possible seating or minor slip effects. Therefore, the apparent post-offset energy absorption should be interpreted as a comparative engineering parameter rather than as the intrinsic fracture energy of the cementitious composite or as energy dissipated exclusively within the material. Because the same testing configuration and calculation procedure were applied consistently to all specimens, this parameter provides a consistent basis for comparing the influence of glass-fiber content on the global tensile response of the investigated composites.

## 3. Results and Discussion

This section presents and analyzes the experimental results obtained in direct tensile tests performed on cement mortar composites reinforced with alkali-resistant glass fibers. The mechanical response is analyzed in terms of stress–strain, apparent initial stiffness, 0.2% offset stress, maximum tensile strength and energy absorption capacity, with special emphasis on the influence of glass fiber content. The results are interpreted by comparing the different reinforcement levels and analyzing the underlying mechanical mechanisms governing the initial response, nonlinear transition behavior and post-cracking deformation. This integrated analysis provides information on how the incorporation of glass fiber modifies the tensile behavior and toughness of cement mortar composites.

### Stress–Strain Response Under Direct Tension

**Figure 4:**
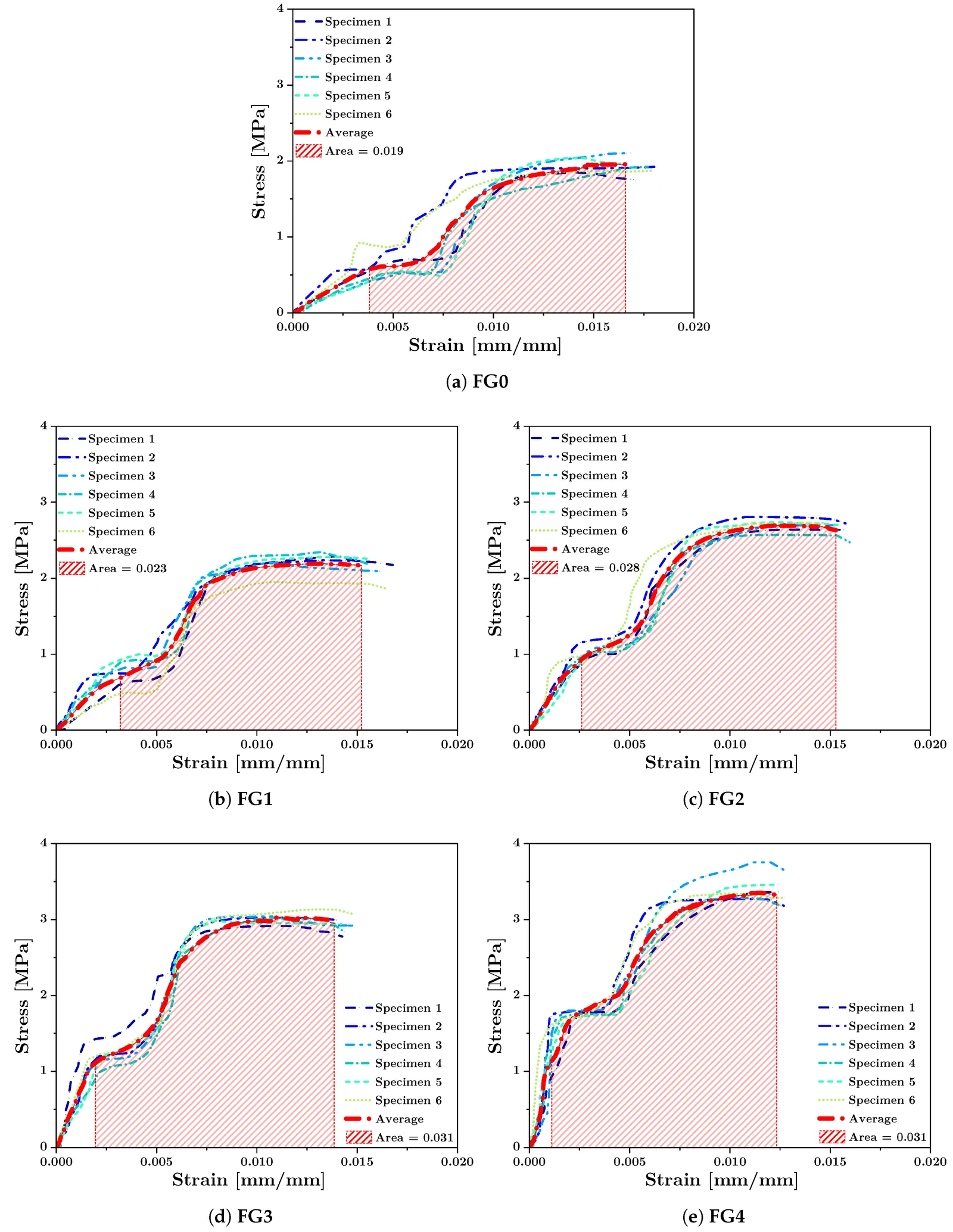
Direct tensile stress–strain (σ–ε) responses of the composite mortar specimens: (**a**–**e**) Individual curves for groups FG0 (0% fiber) through FG4 (10% fiber). Each group comprises six specimens (n=6) cut from a single parent plate. Bold dashed lines indicate the corresponding group-average curves. Shaded regions denote the apparent post-offset energy absorption calculated from the 0.2% offset stress to the end of the recorded response.

Figure 4 shows the experimental (σ−ε) curves obtained from direct tensile tests for each glass fiber content considered FG[%]={0;4;6;8;10}. For each group, the individual stress–strain responses of the six specimens are presented, together with their corresponding average curve. This representation allows for direct evaluation of both the variability between specimens and the representative mechanical behavior of each composite configuration. The (σ−ε) curves exhibit a multi-stage response characterized by an initial approximately linear regime, followed by a nonlinear transition and finally a post-offset region associated with progressive degradation of the load-carrying capacity of the composite system.

The experimental results show acceptable repeatability within each fiber-content group (Figure 4), although the degree of variability differs among the evaluated mechanical parameters. The individual stress–strain curves show a high degree of agreement, and the average curve closely follows the response of all specimens, indicating good repeatability and consistency in the manufacturing and testing procedures. The observed repeatability supports the consistency of the specimen fabrication and testing procedures; however, the variability differs depending on the mechanical parameter being evaluated. The FG0 specimens exhibited the lowest stiffness and strength among all tested configurations. Nevertheless, the sustained response measured after the initial nonlinear transition is consistent with continued load transfer through the embedded galvanized steel mesh and the fiber-reinforced mortar. The stress–strain response was characterized by an approximately linear initial region followed by post-cracking deformation at relatively low stress levels. However, the composite has low stiffness, characterized by a relatively linear elastic response followed by post-cracking deformation due to low stress. In contrast, the incorporation of alkali-resistant glass fibers progressively modifies the tensile response, resulting in greater stiffness and a more gradual transition from the initial linear response to the post-offset regime. As the fiber content increases from 4% to 10%, both the 0.2% offset stress and the ultimate tensile strength show a clear upward trend, which is consistent with an increased load-transfer capacity provided by the incorporated glass fibers.

In addition, the fiber-reinforced specimens exhibited a more sustained global tensile response beyond the 0.2% offset point compared with the reference group. This behavior is consistent with the contribution of fibers to load transfer across cracked regions, which may restrict crack opening and promote a more distributed stress field within the composite [63]. This trend was particularly evident at fiber contents of 8% and 10%, where higher stress levels were sustained beyond the 0.2% offset point. However, because crack initiation locations, crack trajectories, and final failure zones were not systematically documented, these local mechanisms cannot be directly confirmed, and the local failure process cannot be fully assessed from the present experimental dataset.

**Figure 5:**
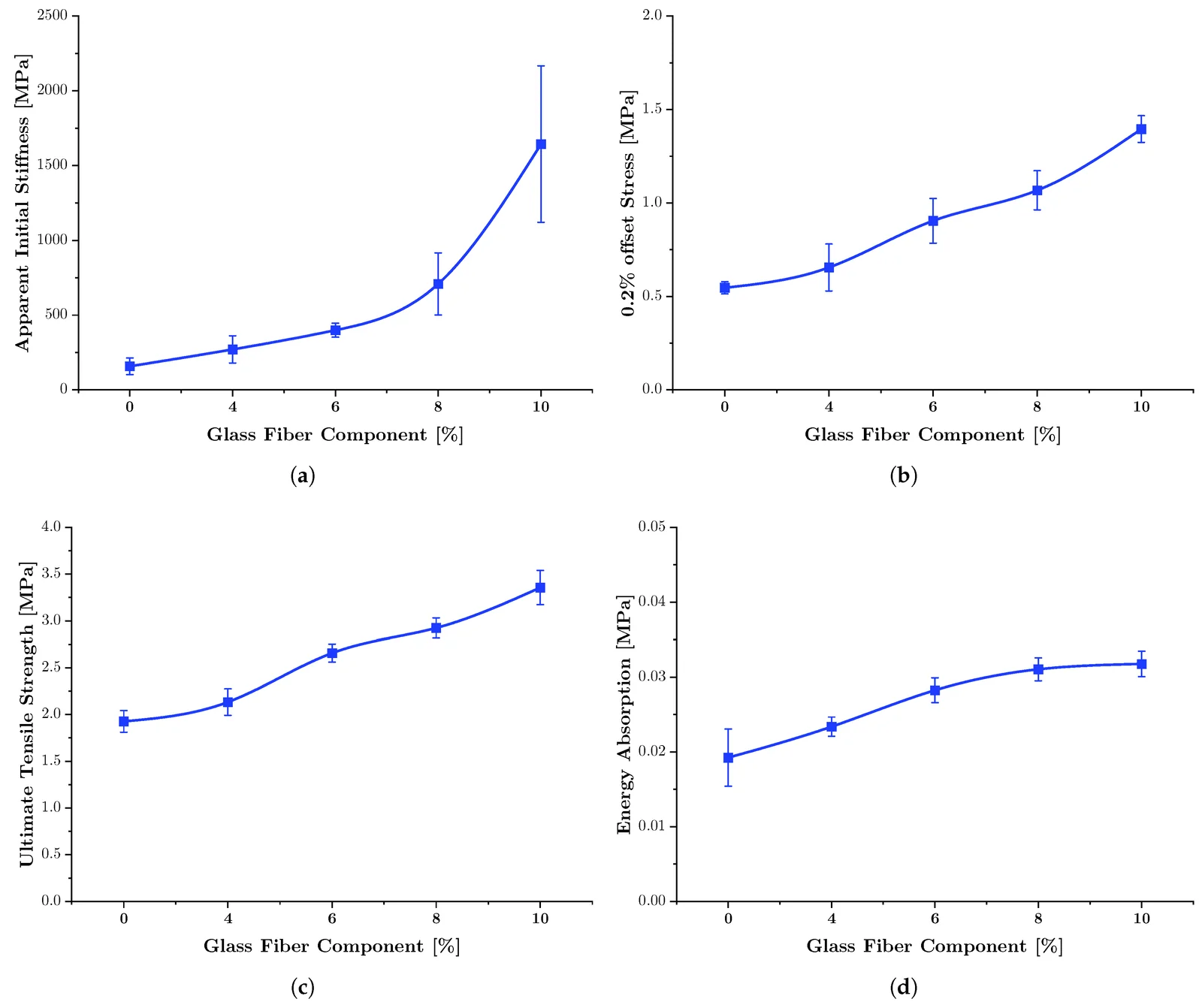
Influence of alkali-resistant glass-fiber content from 0% to 10% relative to the cement volume on the global tensile-response parameters of the steel mesh-reinforced mortar specimens: (**a**) apparent initial tensile stiffness; (**b**) 0.2% offset stress; (**c**) ultimate tensile strength; and (**d**) apparent post-offset energy-absorption density calculated from the operational offset point to the end of the recorded response. Data points represent the mean values obtained from six specimens cut from one parent plate for each fiber content, and error bars indicate ±1 standard deviation.

To quantify the macro-mechanical response, Figure 5 illustrates the evolutionary trends of the tensile properties, including apparent initial tensile stiffness, the characteristic stress determined using the adopted 0.2% offset criterion, ultimate tensile strength, and apparent energy absorption, as a function of AR-glass-fiber content. To complement these visual trends and quantify the specimen-to-specimen variability within each fiber-content group, Table 2 reports the corresponding mean values, standard deviations, and coefficients of variation (COV (%)). The results show increasing trends in the evaluated response parameters across the investigated fiber-content groups, indicating a positive association between alkali-resistant glass-fiber content and the global initial and post-offset tensile response of the steel mesh-reinforced mortar composite within the present experimental program.

As shown in Figure 5a, the mean crosshead-derived apparent initial tensile stiffness was 157.68 MPa for the reference group without glass fibers and 1644.37 MPa at 10% glass-fiber content; thus, the latter mean was 10.43 times the reference-group mean. This ratio characterizes the global tensile response derived from crosshead displacement and should not be interpreted as a 10.43-fold increase in the intrinsic material stiffness. It should be emphasized, however, that this parameter was obtained from crosshead displacement measurements and therefore reflects the global response of the specimen–machine system rather than the intrinsic elastic stiffness of the material. Previous studies on glass fiber-reinforced cementitious mortars have generally reported only modest increases in the elastic modulus, typically below 2%, indicating that the incorporation of dispersed glass fibers alone has a limited influence on the intrinsic elastic stiffness of the cementitious matrix [64]. Therefore, the substantially higher increase observed in the present study should not be directly compared with conventional elastic modulus measurements. Instead, it is likely associated with the combined contribution of the alkali-resistant glass fibers, the embedded galvanized steel mesh, and the tensile test configuration based on crosshead displacement. Consequently, the apparent tensile stiffness reported herein should be interpreted as a structural response parameter specific to the tested composite configuration rather than as an intrinsic material property.

It should be noted that the magnitude of the apparent stiffness increase observed in the present study is influenced by the methodology adopted for strain estimation. Since engineering strain was calculated from the crosshead displacement recorded by the testing machine, the reported stiffness values represent the global response of the specimen–machine system rather than the intrinsic elastic modulus of the mortar composite. Consequently, factors such as seating effects at the grips, compliance of the loading system, and the interaction between the galvanized steel mesh and the fiber-reinforced mortar may contribute to the measured stiffness response. In addition, the measured evolution with increasing fiber content is consistent with crack-bridging and stress-redistribution mechanisms within the composite, contributing to a more stable global tensile response. Therefore, the substantial increase observed in apparent tensile stiffness should be interpreted as a combined effect of the reinforcement mechanisms and the adopted displacement-based measurement procedure rather than as a direct increase in the intrinsic Young’s modulus of the cementitious matrix.

Figure 5b shows the characteristic stress determined using the adopted 0.2% offset criterion. The mean value increased from 0.55 MPa for FG0 to 1.40 MPa for FG4, corresponding to an increase of approximately 154.5% under this operational convention. This result indicates that the intersection between the measured global response and the adopted offset line occurred at progressively higher stress levels as the alkali-resistant glass-fiber content increased. Because alternative offset values were not evaluated, the reported increase should therefore be interpreted as a criterion-specific comparative parameter and not as evidence of a distinct material yielding point.

The ultimate tensile strength results, presented in Figure 5c, show a steady increase with increasing glass-fiber content. The mean ultimate tensile strength increased from 1.93 MPa for FG0 to 3.36 MPa for FG4, corresponding to an improvement of approximately 74.1% at 10% glass-fiber content. This trend is consistent with the beneficial influence of alkali-resistant glass fibers commonly reported for cementitious composites and may be associated with crack-bridging and stress-redistribution mechanisms, which can contribute to sustaining tensile load as cracking develops. Similar improvements in the tensile performance of glass fiber-reinforced mortars were reported by Małek et al. [64]. However, because their study evaluated tensile behavior using splitting tensile tests rather than direct tensile loading, a direct quantitative comparison is not appropriate. Nevertheless, the substantial increase measured in the present study demonstrates that the combination of randomly incorporated alkali-resistant glass fibers and an embedded galvanized steel mesh effectively enhances the tensile load-carrying capacity of the tested composite system.

Figure 5d shows the apparent energy absorption calculated from the characteristic point determined using the adopted 0.2% offset criterion to the end of the recorded response. The mean value increased from 0.019 MPa for FG0 to 0.032 MPa for FG4, corresponding to an increase of approximately 68.4% at 10% glass-fiber content. This result indicates that increasing the glass-fiber content was associated with a larger global stress–strain area after the operational offset point and, consequently, with greater apparent post-offset damage tolerance under the adopted testing configuration. Because the lower integration limit depends on the selected 0.2% offset criterion, the reported value should be interpreted as a criterion-specific comparative parameter rather than as an intrinsic fracture energy.

Among the mechanical properties evaluated, the maximum tensile strength exhibited the lowest variability and, therefore, the highest repeatability. In contrast, the initial apparent tensile stiffness showed comparatively higher coefficients of variation. This greater dispersion is expected, since the apparent stiffness was derived from the initial portion of the stress–strain response using measurements of the the displacement of moving head, making it more sensitive to sample heterogeneity, settling effects in the grips, the flexibility of the testing system, and the random distribution of glass fibers. Consequently, the variability should be interpreted in light of the specific mechanical parameter being evaluated, rather than as a uniform characteristic of the entire experimental program.

Although six specimens were tested for each fiber content, all specimens within a given group originated from the same plate. Therefore, the reported coefficients of variation quantify specimen-to-specimen variability within a manufacturing batch rather than variability between independent production batches.

The apparent post-offset energy-absorption values reported in Figure 5d correspond to the shaded areas shown in the global stress–strain responses in Figure 4. Because these responses were constructed using crosshead displacement, both the apparent initial tensile stiffness and the apparent post-offset energy absorption reflect the global response of the specimen–grip–machine system and may include contributions from machine and grip compliance, seating, and possible minor slip effects. These quantities should therefore be interpreted as comparative global-response parameters rather than as intrinsic material properties. Because the same specimen geometry, gripping arrangement, and loading procedure were applied consistently to all specimens, the observed trends provide a consistent basis for comparing the investigated glass-fiber contents.

Overall, the results presented in Table 2 demonstrate systematic increases in the global tensile-response parameters with increasing alkali-resistant glass-fiber content. At a glass-fiber content of 10% relative to the cement volume, the mean crosshead-derived apparent initial tensile stiffness at 10% glass-fiber content was 10.43 times the reference-group mean, while the 0.2% offset stress, ultimate tensile strength, and apparent post-offset energy absorption increased by 154.5%, 74.1%, and 68.4%, respectively, relative to the FG0 reference configuration. These consistent trends indicate a positive association between increasing alkali-resistant glass-fiber content and higher global tensile-response parameters across the investigated fiber-content groups.

## 4. Conclusions

This study systematically evaluated the direct tensile response of thin galvanized steel mesh-reinforced cement mortar plates containing volumetric fractions of randomly incorporated alkali-resistant glass fibers ranging from 0% to 10% relative to the cement volume. The experimental results showed progressively higher global tensile-response parameters across the investigated glass-fiber-content groups.

At a glass-fiber volumetric fraction of 10%, the mean crosshead-derived apparent initial tensile stiffness was 10.43 times that of the steel mesh-reinforced reference group without glass fibers. The mean 0.2% offset stress was 154.5% higher than that of the reference group, indicating that the intersection between the measured response and the adopted offset line occurred at progressively higher stress levels across the investigated fiber-content groups.

The mean ultimate tensile strength was 74.1% higher at 10% glass-fiber content than in the reference group, while the mean apparent post-offset energy absorption was approximately 68.4% higher. The simultaneous increases in crosshead-derived apparent initial tensile stiffness, 0.2% offset stress, ultimate tensile strength, and apparent post-offset energy absorption indicate a consistent positive association between increasing nominal glass-fiber content and higher global tensile-response parameters across the investigated fiber-content groups. Because strain was estimated from crosshead displacement, the apparent stiffness and energy-absorption values represent comparative global-response parameters of the specimen–grip–machine system rather than intrinsic material properties.

These findings support the continued development of the investigated composite configuration for thin cementitious elements requiring improved tensile response and energy absorption. The present results are limited to the specific mortar composition, reinforcement configuration, manufacturing procedure, and quasi-static direct tensile conditions investigated. In addition, crack initiation locations and final failure zones were not systematically documented; therefore, the local failure process cannot be fully assessed from the present study. Future work should include testing of independently manufactured batches, quantitative characterization of fiber orientation and spatial distribution, analysis of crack development and local failure mechanisms, and evaluation under long-term, cyclic, and environmental loading conditions.

## Figures and Tables

**Table 1:** Specimen groups, glass fiber content, and mix proportions used in the tensile tests.

Specimen ID	Glass Fiber [%]	N° SPECIMENS	Cement [g]	Coarse Sand [g]	Medium Sand [g]	Fine Sand [g]	Water [mL]	AR Glass Fiber [g]
FG0	0	6	500	500	500	500	250	0.00
FG1	4	6	500	500	500	500	250	17.14
FG2	6	6	500	500	500	500	250	25.71
FG3	8	6	500	500	500	500	250	34.29
FG4	10	6	500	500	500	500	250	42.86

**Table 2:** Statistical summary of the tensile properties obtained for each glass fiber content.

Property	FG [%]	Mean	SD	COV [%]
Apparent Initial Stiffness [MPa]	FG0	157.68	55.86	35.43
	FG1	270.78	91.22	33.69
	FG2	399.35	46.94	11.75
	FG3	708.57	185.66	26.20
	FG4	1644.37	523.33	31.83
0.2% Offset Stress [MPa]	FG0	0.55	0.03	5.96
	FG1	0.65	0.13	19.17
	FG2	0.90	0.12	13.20
	FG3	1.07	0.10	9.80
	FG4	1.40	0.07	5.13
Ultimate Tensile Strength [MPa]	FG0	1.93	0.12	6.02
	FG1	2.13	0.14	6.62
	FG2	2.66	0.10	3.61
	FG3	2.92	0.11	3.67
	FG4	3.36	0.18	5.46
Apparent Post-Offset Energy- Absorption Density [MPa]	FG0	0.019	0.004	19.83
	FG1	0.023	0.001	5.50
	FG2	0.028	0.002	5.91
	FG3	0.031	0.002	4.91
	FG4	0.032	0.002	5.37

## Data Availability

The original contributions presented in this study are included in the article. Further inquiries can be directed to the corresponding authors.

## References

[1] Parghi A., Alam M.S. (2018). A review on the application of sprayed-FRP composites for strengthening of concrete and masonry structures in the construction sector. Compos. Struct..

[2] Danieli S., Neto J.S.A., Soares E.G., Oliveira T.F., Brito B.L., Kirchheim A.P. (2025). Shaping a sustainable path: Exploring opportunities and challenges in carbon capture and utilization in cement and concrete industry. CEMENT.

[3] Ülger T., Cavuslu M. (2025). Durability and flexural behavior of concrete-filled hybrid GFRP beams with various interface treatments under humid environmental exposure. Case Stud. Constr. Mater..

[4] Garg M., Azarsa P., Gupta R. (2021). Self-Healing Potential and Post-Cracking Tensile Behavior of Polypropylene Fiber-Reinforced Cementitious Composites. J. Compos. Sci..

[5] Fernando V., Bastola R., Lokuge W., Gunasekara C., Wang H. (2025). Flexural behaviour, cracking, and serviceability of sustainable RC beams with waste glass fine aggregate and pond ash. Eng. Struct..

[6] Chen B., Wang Y., Peng L., Zhao Y. (2025). Durability performance of a seawater fully recycled aggregate concrete (SFRAC) frame structure under four-year marine exposure. Eng. Struct..

[7] Ahmed H.U., Faraj R.H., Hilal N., Mohammed A.A., Sherwani A.F.H. (2021). Use of recycled fibers in concrete composites: A systematic comprehensive review. Compos. Part B Eng..

[8] Paul S.C., van Zijl G.P., Šavija B. (2020). Effect of Fibers on Durability of Concrete: A Practical Review. Materials.

[9] Moein M.M., Moein A.M., Saradar A., Rigby S.E., Tazari H., Karakouzian M. (2025). Mechanical properties of Portland cement concrete mixed with different doses of recycled brick powder and steel fiber. Heliyon.

[10] Hu X., Xu H., Xi X., Zhang P., Yang S. (2022). Meso-scale phase field modelling of reinforced concrete structures subjected to corrosion of multiple reinforcements. Constr. Build. Mater..

[11] Akbulut Z.F., Guler S. (2025). Structural self-compacting lightweight concrete: Effects of fly ash and basalt fibers on workability, thermal and mechanical properties under ambient conditions and high temperatures. Constr. Build. Mater..

[12] Miarka P., Ríos J.D., Malíková L., Bílek V. (2026). Fatigue resistance of concrete revisited: Wide range data from low-cycle to high-cycle and influence of aggregate on fatigue lifetimes. Cem. Concr. Compos..

[13] Xiong S., Zhang H., Liu W., Zhang L. (2026). Resolving the strength-permeability trade-off in recycled aggregate pervious concrete by fiber reinforcement. J. Build. Eng..

[14] Ahmad W., Khan M., Smarzewski P. (2021). Effect of Short Fiber Reinforcements on Fracture Performance of Cement-Based Materials: A Systematic Review Approach. Materials.

[15] dos Santos V.B., de Oliveira V.M., Krahl P.A., de Andrade Silva F., Cardoso D.C.T., Rossi A., Martins C.H. (2025). Flexural behavior of steel-ultra-high performance concrete composite beams under negative bending with varying fiber contents. J. Build. Eng..

[16] Hung C.C., Chen S.E., Tsai Y.J., Yen C.H. (2026). Impact of fiber-induced tensile strain hardening on the bearing performance of ECC and UHPC pipes: Experimental study and strength modeling. Case Stud. Constr. Mater..

[17] Zhang P., Yang Y., Wang J., Jiao M., Ling Y. (2020). Fracture Models and Effect of Fibers on Fracture Properties of Cementitious Composites—A Review. Materials.

[18] Hassanpour M., Shafigh P., Mahmud H.B. (2012). Lightweight aggregate concrete fiber reinforcement—A review. Constr. Build. Mater..

[19] Mani S., Partheeban P., Gifta C.C. (2025). A comprehensive review on multilayered natural-fibre composite reinforcement in geopolymer concrete. Clean. Mater..

[20] Yang S., Chen Z., Chen H., Lan T. (2025). Evaluation of Tensile Strength and Fracture Toughness of Cement Mortar with Different Sand Fineness Moduli Based on Boundary Effect Model. J. Mater. Civ. Eng..

[21] Subandi, Yatnikasari S., Damaiyanti M., Azzahra R., Vebrian (2019). Effect of additional fiberglass fiber on concrete performance. Ann. Chim. Sci. Mater..

[22] Luo Y., Lin G., Chen J.F., Wu J. (2025). Thermoplastic fiber-reinforced polymer composites in construction: A state-of-the-art review. Case Stud. Constr. Mater..

[23] Xiaochun Q., Xiaoming L., Xiaopei C. (2017). The applicability of alkaline-resistant glass fiber in cement mortar of road pavement: Corrosion mechanism and performance analysis. Int. J. Pavement Res. Technol..

[24] Zhang P., Qin Z., Gao Z., Wang F., Lai C. (2025). Research progress on impact resistance of fiber-reinforced concrete—A review. J. Build. Eng..

[25] Feng Q., Hu W., Liu L., Luo J. (2025). State-of-Art on Workability and Strength of Ultra-High-Performance Fiber-Reinforced Concrete: Influence of Fiber Geometry, Material Type, and Hybridization. SDHM Struct. Durab. Health Monit..

[26] Manso-Morato J., Hurtado-Alonso N., Revilla-Cuesta V., Skaf M., Ortega-López V. (2024). Fiber-Reinforced concrete and its life cycle assessment: A systematic review. J. Build. Eng..

[27] Ma W., Zheng J., Feng S., Yuan Z., Zhong Q., Guo S. (2026). Influence mechanism of alkaline activators on the high temperature resistance of steel fiber-reinforced supersulfated cement mortar. J. Build. Eng..

[28] Du Y., Wang Z., Bai Q., Huang H., Bai E. (2026). Investigation on the mechanical properties of polypropylene fiber-reinforced sulphoaluminate cement mortar under negative temperatures and development of an Intelligent predictive model. J. Build. Eng..

[29] Ezziane M., Bouzouaid S., Sahraoui M., Messaoudene I., Hani M., Chetbani Y., Belaadi A., Alshaikh I.M., Alnmr A., Ghernaout D. (2025). Residual performance of Portland cement types-based plain and steel fibre-reinforced mortars exposed to elevated temperatures. Results Eng..

[30] Delavar M.R., Aslani F., Sercombe T. (2025). Cracking behaviour in 3D concrete printed fibre-reinforced cementitious composites: A review. J. Build. Eng..

[31] Xu K., Huang W., Wang Y., Chen Q., Ju J.W., Zhu H. (2025). Fracture process in steel fiber-reinforced concrete: A 3D mesoscale modeling study. Constr. Build. Mater..

[32] Abbass W., Khan M.I., Mourad S. (2018). Evaluation of mechanical properties of steel fiber reinforced concrete with different strengths of concrete. Constr. Build. Mater..

[33] Du W., Yu F., Qiu L., Guo Y., Wang J., Han B. (2024). Effect of Steel Fibers on Tensile Properties of Ultra-High-Performance Concrete: A Review. Materials.

[34] Valeri P., Fernàndez Ruiz M., Muttoni A. (2020). Tensile response of textile reinforced concrete. Constr. Build. Mater..

[35] Wu G., An Q., Li H., Mou D. (2023). Physical and mechanical properties of cotton straw fibre and expanded polystyrene cementitious composite. Adv. Cem. Res..

[36] Shuling G., Jiayao Z., Yanping Z., Zhe W. (2022). Mechanics behaviors study of Hybrid fiber reinforced cementitious composites exposed high temperatures. Adv. Cem. Res..

[37] Guo S., Yu T., Mao K., Cao P., Lu J., Zhong H. (2025). Study on fracture properties and damage of glass fiber reinforced concrete under corrosive environment. Constr. Build. Mater..

[38] Gao F.F., Xu Z.T., Wang W.D., Zhao Y., Shi Y.L. (2025). Interfacial bonding strengthening mechanisms in recycled GFRP waste fibers-ultra high performance concrete through synergistic nano-silica/silane. Constr. Build. Mater..

[39] Zhou B., Zhang M., Ma G., Zhang R. (2025). Evaluation of mechanical properties, durability, and sustainability of concrete reinforced with recycled GFRP fibers: Experiments and optimization. J. Build. Eng..

[40] Chen H., Wang P., Pan J., Lawi A., Zhu Y. (2021). Effect of alkali-resistant glass fiber and silica fume on mechanical and shrinkage properties of cement-based mortars. Constr. Build. Mater..

[41] Xin Q., Li Z., Lu S., Shao P., Zhang M. (2024). The synergistic effect of recycled glass fiber reinforced plastic and silica fume on cement mortar properties. J. Build. Eng..

[42] Fenu L., Forni D., Cadoni E. (2016). Dynamic behaviour of cement mortars reinforced with glass and basalt fibres. Compos. Part B Eng..

[43] Zaid O., Kahla N.B. (2026). Natural fiber reinforcements in cement-based composites: A review on recent advances, properties, and sustainability. Ind. Crops Prod..

[44] Demirdağ C., Nodehi M., Bideci A., Bideci Ö.S., Tuncer M., Gencel O., Ozbakkaloglu T. (2024). The use of natural (coconut) and artificial (glass) fibers in cement–polymer composites: An experimental study. Constr. Build. Mater..

[45] Zainal S.M.S., Abel M., Durai D., Rizalman A.N., Sulaiman M.F. (2024). Experimental investigation on the mechanical properties of cement mortar containing recycled tire crumbs reinforced with polypropylene, oil palm and banana fibers. J. Build. Eng..

[46] Devi C., Vijayan D., Nagalingam R., Arvindan S. (2022). A review of the implementations of glass fiber in concrete technology. Mater. Today Proc..

[47] Dawood E.T., Shawkat A.S., Abdullah M.H. (2021). Flexural performance of ferrocement based on sustainable high-performance mortar. Case Stud. Constr. Mater..

[48] Awani O., El-Maaddawy T., Ismail N. (2017). Fabric-reinforced cementitious matrix: A promising strengthening technique for concrete structures. Constr. Build. Mater..

[49] Zhou F., Liu H., Du Y., Liu L., Zhu D., Pan W. (2019). Uniaxial Tensile Behavior of Carbon Textile Reinforced Mortar. Materials.

[50] Deng M., Dong Z., Zhang C. (2020). Experimental investigation on tensile behavior of carbon textile reinforced mortar (TRM) added with short polyvinyl alcohol (PVA) fibers. Constr. Build. Mater..

[51] Wang J., Yu D., Mu F., Ji X., Peng Y. (2023). Toughening Mechanism of Directional Fabric Woven Net and/or Non-Directional Short-Cut Fiber-Reinforced Sprayed Cement Mortar Thin-Plates. Materials.

[52] Garrido C., Pincheira G., Valle R., Fernández J., Tuninetti V. (2024). Plastic deformation behavior and energy absorption performance of a composite metamaterial based on asymmetric auxetic lattices. Compos. Struct..

[53] Garrido C., Fernández J., Tuninetti V., Pincheira G., Ríos I., Valle R. (2025). Resin-Reinforced Auxetic Structures with Re-Entrant Struts for Improved Energy Absorption. Polymers.

[54] Valle R., Garrido C., Tuninetti V. (2026). Programming Failure Mode Transitions in Polyurea-Reinforced 3D-Printed ABS and PA-GF Cellular Metamaterial Composites. Polymers.

[55] Garrido C., Tuninetti V., Valle R. (2026). Triple-hybridization strategy for crashworthiness in polyurea-reinforced carbon-fiber/polyamide lattice structures. Thin-Walled Struct..

[56] (2024). Standard Specification for Portland Cement.

[57] (2025). Standard Test Method for Sieve Analysis of Fine and Coarse Aggregates.

[58] Ghalla M., Bazuhair R.W., Mlybari E.A., Mahmoodzadeh A., Elsamak G., Yehia S.A. (2026). Shear performance of unreinforced masonry walls with door and window openings strengthened using welded steel mesh. Sci. Rep..

[59] Buschow K.H.J., Cahn R.W., Flemings M.C., Ilschner B., Kramer E.J., Mahajan S. (2001). Encyclopedia of Materials: Science and Technology.

[60] (2025). Standard Test Method for Tensile Properties of Polymer Matrix Composite Materials.

[61] Fernández J., Garrido C., Muñoz L., Nuñez F., Valle R., Tuninetti V. (2025). Metamortar Composites Reinforced with Re-Entrant Auxetic Cells: Mechanical Performance and Enhanced Energy Absorption. Polymers.

[62] Valenzuela M., Ciudad G., Cárdenas J.P., Medina C., Salas A., Oñate A., Pincheira G., Attia S., Tuninetti V. (2024). Towards the development of performance-efficient compressed earth blocks from industrial and agro-industrial by-products. Renew. Sustain. Energy Rev..

[63] Wu C., Leung C.K., Li V.C. (2018). Derivation of crack bridging stresses in engineered cementitious composites under combined opening and shear displacements. Cem. Concr. Res..

[64] Małek M., Jackowski M., Łasica W., Kadela M., Wachowski M. (2021). Mechanical and Material Properties of Mortar Reinforced with Glass Fiber: An Experimental Study. Materials.

